# Congenital Diaphragmatic Hernia with Gastric Volvulus and Splenic Herniation: An Unusual Delayed Presentation in a Six-Month Child

**DOI:** 10.24248/eahrj.v6i1.674

**Published:** 2022-07

**Authors:** Mathayo Shadrack, Jamil Suleiman, David Msuya, Rune Philemon, Adnan Sadiq, Murad Tarmohamed, Jay Lodhia

**Affiliations:** a Department of General Surgery, Kilimanjaro Christian Medical Center, Moshi, Tanzania; b Kilimanjaro Christian Medical University College, Faculty of Medicine, Moshi, Tanzania; c Department of Pediatrics, Kilimanjaro Christian Medical Center, Moshi, Tanzania; d Department of Radiology, Kilimanjaro Christian Medical Center, Moshi, Tanzania

## Abstract

**Background:**

Acute gastric volvulus associated with congenital diaphragmatic hernia is an uncommon disorder in infancy and a surgical emergency.

**Methods:**

We present a six-month female baby who presented with clinical features of intestinal obstruction. Ultrasonography of the abdomen revealed gastric volvulus. The baby underwent emergency laparotomy. Gastric volvulus with splenic herniation was encountered through a diaphragmatic hernia.

**Results:**

The defect was corrected, the stomach and spleen were mobilized into the normal anatomical position. The baby recovered well.

**Conclusion:**

A high index of clinical suspicion and thorough radiological assessments are necessary for this life-threatening condition along with surgical correction of the abnormalities.

## BACKGROUND

Congenital Diaphragmatic Hernia (CDH) is a developmental abnormality due to the incomplete fusion and closure of the pleuroperitoneal canal during fetal development with an incidence of 0.8/10,000 to 5/10,000 births^1,2^. CDH can be diagnosed prenatally or, as is often the case, during the neonatal period. Occasionally, CDH can be missed, and children can present later in acute respiratory or gastrointestinal symptoms.^1,3^

Infants often present with respiratory distress, and presentation after infancy occurs in 5% to 10% of the affected neonates and present with respiratory distress from pleural effusion or gastrointestinal distress from intestinal obstruction. However, 1% of the cases can be asymptomatic and discovered incidentally on imaging.^4^ Despite the advances in medical and surgical management of CDH the morbidity and mortality remains high.^2^ Gastric volvulus associated with CDH constitutes a surgical emergency. We present a six-month-female from Tanzania with acute gastric volvulus associated with CDH who presented with features of gastrointestinal obstruction.

## CASE PRESENTATION

A six-month-old female baby was referred to our tertiary centre from a regional hospital due to vomiting for four days, inability to pass stool and abdominal distention for three days. The vomiting was projectile and bilious but not stained with blood. There was no fever reported. Herbal medication was given at home with no improvement. The parents are Maasai who are the indigenous tribe, and the baby was born at term by SVD at a health centre with a birth weight of 2900 grams and received all immunisations according to local guidelines. On general examination, the patient was sick looking, not pale, acyanotic and not dyspneic. Her blood pressure was 110/60 mmHg, pulse rate 125 beats per minute, respiration rate 28 breaths per minute and saturating at 96% on room air. Body weight on admission was 8700 grams. On systemic examination, the abdomen was distended more on the left upper quadrant with traditional markings on the upper quadrants. The abdomen on palpation was tense and tender more at the epigastric region, and bowel sounds were reduced on auscultation. The rectum was empty on digital rectal examination. Heart sounds were heard normally on auscultation and lungs revealed reduced air entry more on the left lower zone.

**FIGURE 1.**
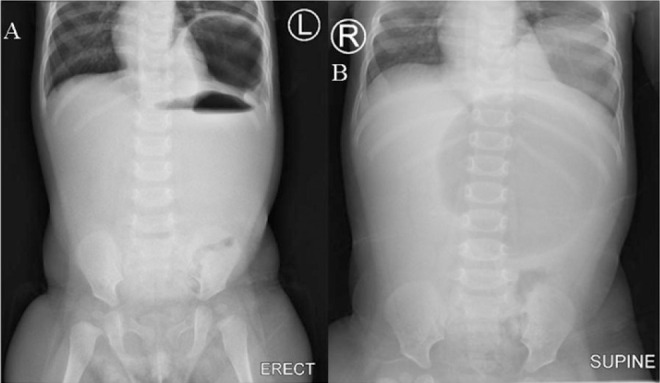
An Abdominal X-Ray showing Gastric Air-Fluid Levels A: Erect abdomen x-ray. B: Supine abdomen x-ray. X-rays demonstrate a left congenital diaphragmatic hernia with protrusion of the stomach into the left hemithorax. Findings suggestive of bochdalek hernia.

Abdominal ultrasound (Siemens Acuson NX3, Germany) was done revealing gastric volvulus and reduced gastric peristalsis. An abdominal x-ray showed gastric air-fluid levels (Figure 1).

Her complete blood count revealed microcytic hypochromic anemia of 8.8 g/dl, raised leukocyte count of 21×10^9^/L and a normal platelet count of 447×10^9^/L. The serum creatinine was normal at 57 μmol/l, serum sodium 180.02 mmol/L, serum potassium 3.51 mmol/L, aspartate aminotransferase 34.02 U/l and alanine aminotransferase 13.72 U/l. Blood group was O Rhesus-positive.

**FIGURE 2.**
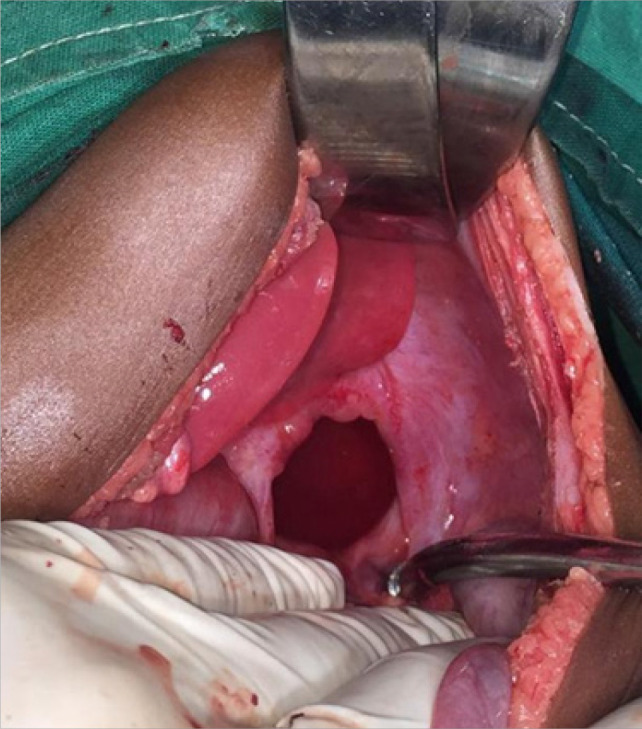
Image Showing Left Sided Diaphragmatic Defect

The patient was given Ceftriaxone 450 mg and Metronidazole 80 mg intravenously and transfused one unit (200 milliliters) of whole blood before being taken for an emergency laparotomy. The abdomen was opened through a transverse incision, the stomach was grossly distended, the gastric antrum was rotated clockwise about 90 degrees at the level of the fundus. There was a left diaphragmatic defect of about 3 × 4 centimeters (Figure 2). The fundus and spleen were both herniated through the defect into the chest. The stomach was mobilized and rotated anticlockwise to its anatomical position. The fundus was reduced back followed by the spleen. Suctioning of fluid content from the chest was done, and the diaphragmatic defect was repaired using nylon suture in a continuous technique. The abdomen was cleaned thoroughly with warm saline and closed in layers.

**FIGURE 3.**
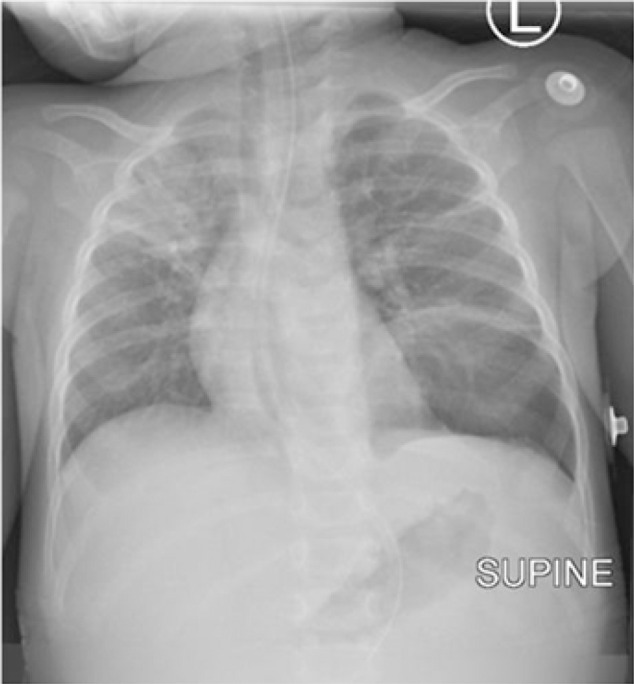
Post-operative Supine Antero-Posterior Chest X-Ray Chest x-ray, no longer shows protruding stomach in the left hemithorax. Consolidation of the anterior segment of the right upper lobe noted in keeping with pneumonia. Atelectatic bands seen in the left mid zone.

Postoperatively, the baby was nursed in ICU for four days, transfused with 180 milliliters of whole blood and kept on intravenous Ceftriaxone 450 mg once daily and Metronidazole 80 mg eight-hourly for five days along with Paracetamol 140 mg for 24 hours. Control chest x-ray was done after 24-hours (Figure 3). Control full blood picture revealed leucocyte count of 9.71×10^9^/L, hemoglobin of 16.9 g/dl and a platelet count of 200×10^9^/L. The baby fared well and was discharged on day six postoperatively with Amoxicillin-Clavulanic acid syrup for five days and to return to the surgical outpatient clinic after two weeks for a review of which the baby fared well, was tolerating meals and incision had healed. After two months the baby was reviewed again whereby the mother reported the baby to continue to gain weight well and reported of no complaints hence was discharged.

## DISCUSSION

A congenital diaphragmatic hernia is a developmental defect characterized by incomplete fusion of the pleuroperitoneal canal, usually evident at birth^1,4^. This defect creates a pathway for intra-abdominal structures to herniate into the thoracic cavity. It is mainly classified anatomically into three main types; posterolateral (Bochdalek) hernia which is the most common type (80-90% of cases), anterior parasternal or retrosternal (Morgagni) hernia and Central hernia.^4^ Our child presented with a posterolateral hernia.

A diaphragmatic defect increases the risk of gastric volvulus. However, this presentation is infrequent, with Javier et al stating that only 26 pediatric cases had been reported worldwide by 2008, and only 15 of the patients were infants.^3^ The risk is increased due to anomalies in any of the four ligaments (gastrocolic, gastrohepatic, gastrophrenic and gastrosplenic) inserted in the diaphragm, that hold the stomach in position^3,5,6^. In patients with a defect in the diaphragm, the gastric fixation can be elongated or absent.^7^ Increased subdiaphragmatic space could also increase the risk of an abnormal stomach rotation.^8^

Gastric volvulus can be classified into three types, organoaxial, mesenteroaxial and combined.^9-11^ Organoaxial volvulus, as seen in our case, is the most common type seen in 60% of volvulus cases. In organoaxial volvulus, the rotation occurs along the cardio-pylorus axis due to abnormal mobility and deficient fixation at the hiatus of the stomach.^12^ A diaphragmatic hernia is frequently associated with this type.^13^ In mesenteroaxial volvulus, the stomach rotates around an axis between the greater and lesser curvatures. Finally, in the combined type, the stomach rotates about both axes.^12^ Anomalies in the ligaments could also lead to splenic herniation as seen in the index case, although it is a rare finding in the postneonatal age.^14^ Some authors report that a mobile spleen could potentially cause the volvulus by drawing the gastric fundus downwards in an erect position.^15^

The classical triad of Borchardt which include epigastric distension, unproductive retching and inability to pass naso-gastric tube is usually described in adults, and rarely seen in neonates and infants^12,16^. Neonates usually have a typical presentation of vomiting and respiratory distress while infants present with vomiting and epigastric distentionas seen in our case.^14,17^

The diagnosis is suspected when an erect chest radiograph shows a high air-fluid level, and abdominal radiographs show increased soft tissue density in the upper abdomen.^18^

Further complimentary tests are needed, such as a CT-scan or barium contrast studies, with the latter serving to confirm the diagnosis. If the diagnosis is evident with a plain X-ray, as seen in our case, surgical management should not be delayed with further tests^1,3^.

Since the diagnosis is based on a high index of suspicion, in some instances, the condition is misdiagnosed as pneumothorax, pleural effusions, hydrothorax, hydropneumothorax, bronchogenic cysts and hence subjected to inappropriate thoracocentesis^19,20^.

Surgical management involves performing a laparotomy, usually as an emergency. It involves detortion of the volvulus, reduction of the herniated structure with or without resection of the necrotic segments, and repairing of the diaphragmatic defect.^3,21-23^ It is recommended to fix the stomach either by gastrostomy or gastropexy, but some authors reported no fixation, with few cases or recurrences.^22,24^ In our case, fixation of the stomach was not required as it was not redundant, and after reduction, it returned to its normal anatomical position.

Acute gastric volvulus with congenital diaphragmatic hernia is a surgical emergency, with mortality rates estimated up to 80% without surgical intervention.^25^ Any delays in surgical intervention can lead to catastrophic complications such as acute pneumothorax, gastrothorax, splenic torsion, appendicitis, incarceration, strangulation, ischemia, perforation, peritonitis and death^26-28^.

## CONCLUSION

CDH with Gastric volvulus is a surgical emergency, and early diagnosis with immediate surgical intervention is required for good outcome. However many patients have limited access to specialized tertiary referral care centres, therefore diagnosis cannot be established and prompt management cannot be offered. This case report highlights the importance of thorough prenatal screening to aid multidisciplinary management plan and helps creates awareness among clinicians to have CDH as their differential diagnosis.

### Ethical Consderations

Ethical approval was obtained from the Department of General Surgery, Kilimanjaro Christian Medical Centre. Written informed consent was obtained from the patient's mother for publication for this case report; additionally, accompanying images have been censored to ensure that the patient cannot be identified. A copy of the consent is available on record.
